# Astrocytic YAP prevents the demyelination through promoting expression of cholesterol synthesis genes in experimental autoimmune encephalomyelitis

**DOI:** 10.1038/s41419-021-04203-8

**Published:** 2021-10-05

**Authors:** Jingjing Zhang, Xingxing Xu, Huitao Liu, Lingting Jin, Xiya Shen, Changnan Xie, Weiwei Xiang, Danlu Yang, Wenjin Feng, Jiaojiao Wang, Mianxian Wang, Tianyingying Dong, Haoyu Qiu, Lihao Wu, Ying Wang, Xu Zhang, Zhihui Huang

**Affiliations:** 1 School of Pharmacy, and Department of Neurosurgery of the Affiliated Hospital, Hangzhou Normal University, Hangzhou, Zhejiang China; 2 School of Basic Medical Sciences, Wenzhou Medical University, Wenzhou, Zhejiang China; 3 Department of Orthopedics (Spine Surgery), The First Affiliated Hospital of Wenzhou Medical University, Wenzhou, Zhejiang China; 4 Department of Neurology, The First Affiliated Hospital of Wenzhou Medical University, Wenzhou, Zhejiang China; 5 Zhejiang Sinogen Medical Equipment Co., Ltd., Wenzhou, Zhejiang China; 6 Phase I Clinical Research Center, Zhejiang Provincial People’s Hospital of Hangzhou Medical College, Hangzhou, Zhejiang China

**Keywords:** Multiple sclerosis, Astrocyte, Multiple sclerosis

## Abstract

Cholesterols are the main components of myelin, and are mainly synthesized in astrocytes and transported to oligodendrocytes and neurons in the adult brain. It has been reported that Hippo/yes-associated protein (YAP) pathways are involved in cholesterol synthesis in the liver, however, it remains unknown whether YAP signaling can prevent the demyelination through promoting cholesterol synthesis in experimental autoimmune encephalomyelitis (EAE), a commonly used animal model of multiple sclerosis characterized by neuroinflammation and demyelination. Here, we found that YAP was upregulated and activated in astrocytes of spinal cords of EAE mice through suppression of the Hippo pathway. YAP deletion in astrocytes aggravated EAE with earlier onset, severer inflammatory infiltration, demyelination, and more loss of neurons. Furthermore, we found that the neuroinflammation was aggravated and the proliferation of astrocytes was decreased in YAP^GFAP^-CKO EAE mice. Mechanically, RNA-seq revealed that the expression of cholesterol-synthesis pathway genes such as HMGCS1 were decreased in YAP^−/−^ astrocytes. qPCR, western blot, and immunostaining further confirmed the more significant reduction of HMGCS1 in spinal cord astrocytes of YAP^GFAP^-CKO EAE mice. Interestingly, upregulation of cholesterol-synthesis pathways by diarylpropionitrile (DPN) (an ERβ-ligand, to upregulate the expression of HMGCS1) treatment partially rescued the demyelination deficits in YAP^GFAP^-CKO EAE mice. Finally, activation of YAP by XMU-MP-1 treatment promoted the expression of HMGCS1 in astrocytes and partially rescued the demyelination and inflammatory infiltration deficits in EAE mice. These findings identify unrecognized functions of astrocytic YAP in the prevention of demyelination through promoting cholesterol synthesis in EAE, and reveal a novel pathway of YAP/HMGCS1 for cholesterol synthesis in EAE pathology.

## Introduction

Multiple sclerosis (MS) is an autoimmune disease of the central nervous system (CNS) that is characterized by inflammation, demyelination and dyskinesia, although the etiology remains elusive [1, 2]. Patients with MS increase year by year, with 2.65 per 1000 in 2013, and a high recurrence rate [3]. Generally, the onset begins after the age of thirty and the prevalence of women is about twice of men. At present, most approved therapies of MS focus on the symptoms of MS, which merely reduces the frequency of relapses, hardly with specific and effective strategies to interfere with the autoimmune progression [4–6].

The most evident features of MS are demyelination and inflammation, besides, axonal loss or damage, and gliosis are also characteristics of MS [7–10]. Immune cells and glial cells, such as microglia, oligodendrocytes, and astrocytes, are thought to participate in the process of MS [11]. However, much more attention has been paid to microglia, immune cells, and oligodendrocytes in MS. Astrocytes, one of the most abundant cell types in the CNS that exert multiple functions, have generally been considered as a secondary player in EAE, and received less attention consequently [7, 12–15]. Nevertheless, recent accumulating evidences have demonstrated the critical roles of astrocytes in MS [16–19]. Astrocytes are activated within demyelinating lesions, contribute to astrocytic scar formation, regulate demyelination and remyelination of the axons and play dual roles in neuroinflammation of MS and EAE [13, 15, 18–22]. However, it remains unclear how astrocytes regulate the neuroinflammation and demyelination during EAE. In the CNS, regeneration of myelin is mediated by oligodendrocyte progenitor cells through affecting the cholesterol biosynthesis pathway [23]. However, in the adult brain, peripheral cholesterols do not cross the blood–brain barrier, thus most cholesterols must be synthesized in the brain [24, 25]. Studies have shown that in the adult brain, cholesterols are mainly synthesized in astrocytes and transported to oligodendrocytes and neurons [18]. Interestingly, recent studies have shown that under the condition of EAE, cholesterol-synthesis genes such as HMG-CoA synthase 1 (HMGCS1) were decreased in astrocytes [18], however, it remains unclear how these cholesterol-synthesis genes are regulated during EAE.

YAP (yes-associated protein) is a critical downstream target of the Hippo signaling pathway, and regulates self-renewal, tissue regeneration, and organ size [26–28]. As a key transcriptional co-factor of the Hippo pathway, YAP is abundant in astrocytes [29, 30]. It has been well known that the Hippo/YAP pathway participates in cell proliferation, differentiation, and survival during physiological and pathological conditions [31], and the core kinases of this pathway include MST1/2 (mammalian aseptic 20 kinase), SAV1 (salvador homolog 1), LATS1/2 and MOB1. When the Hippo signaling is on, MST1/2 and SAV1 are activated and then phosphorylate and activate LATS1/2, thereby, phosphorylates YAP, resulting in YAP retention and degradation in the cytoplasm [32]. When the Hippo pathway is off, the suppression of LATS1/2 enables unphosphorylated YAP to translocate into the nucleus, where it interacts with DNA binding transcription factors such as TEAD and Smads, to initiate the transcription of multiple genes that participate in cell proliferation, differentiation, and so on [32, 33]. YAP signaling has been reported to promote astrocytes proliferation and differentiation [34, 35], and is crucial for Schwann cell myelination in the peripheral nervous system during development and Schwann cell remyelination after nerve injury [36, 37] and inflammation [29, 38]. Interestingly, recent studies have shown that in the liver, YAP stimulates the transcription of the fatty acid synthase and 30-hydroxymethyl glutaryl coenzyme A reductase (HMGCR), as a consequence, promoting hepatocyte lipogenesis and the synthesis of cholesterols [39]. LATS2 suppresses sterol regulatory element-binding proteins (SREBP) and inhibits hepatic cholesterol accumulation [40]. However, it remains poorly understood about the functions of YAP signaling in EAE, and whether YAP signaling can prevent the demyelination through promoting cholesterol synthesis in EAE.

In the present study, we found that YAP was activated in astrocytes of the spinal cords of the EAE mice, and the knockout of astrocytic YAP exacerbated EAE. Moreover, astrocytic YAP prevented the demyelination through inducing the expression of the cholesterol-synthesis genes such as HMGCS1 in EAE mice. Our findings provide some evidences for the mechanisms underlying demyelination and neuroinflammation that are regulated by astrocytes in EAE, which may contribute to developing novel MS therapy.

## Materials and methods

### Animals

YAP^GFAP^-CKO mice were prepared by crossing the GFAP-Cre mice (from The Jackson Laboratory) with floxed YAP allele (YAP^f/f^). These mice were maintained in the C57BL/6 background, and genotyping was conducted with PCR. YAP^f/f^ mice were described as previously [41].

### Preparation of the EAE model and treatment

The sample size was chosen based on previous experience with the EAE models and studies previously published. The protocol of the EAE model is described as previously [42]. Briefly, 8–10-weeks-old female mice were anesthetized and immunized with 0.2 mg of emulsified MOG_35-55_ (a peptide encoded 35–55 aa of myelin oligodendrocyte glycoprotein) (1:1) [GL Biochem (Shanghai)] subcutaneously in Freund’s complete adjuvant containing 8 mg/ml *Mycobacterium tuberculosis* (strain H37RA; Difco, USA). 300 ng of pertussis toxin (516561, Sigma) was injected at day 0 and day 2 after immunization intraperitoneally. Control mice were immunized in the same manner by PBS in the absence of peptide. From days 0 to 21 after immunization, the body weight of each mouse was daily recorded and the behavioral function was evaluated by the five-point scoring criteria (the scores of EAE ≥ 1 indicated the successful establishment of EAE models) [42].

XMU-MP-1 (HY-100526, MedChemExpress) was dissolved in DMSO and injected intraperitoneally with 1 mg/kg, given every 2 d [41] after EAE induction. For DPN (ERβ-ligand) treatment, DPN (HY-12452, MedChemExpress) was first dissolved in 10% DMSO, and then dissolved in 90% corn oil, reducing the final ethanol concentration to 10%. DPN was initiated 1 week before EAE induction and given through subcutaneous injections every other day at the concentration of 8 mg/kg per day [43]. Each mouse was distributed into experimental groups randomly. Evaluation of genotype and experimental condition was blind.

### Hematoxylin–eosin (HE) staining

The HE staining was conducted as a protocol of HE Staining Kit (G1120, Solarbio). Briefly, the sections (20 μm-thick) were stained with hematoxylin for 1 min and then soaked in the acidic liquid alcohol differentiation for 30 s. After staining with eosin for 50 s and dehydrated by ethanol (95%, 100%), the sections were finally cleared by xylene and mounted. The images were obtained by Ci-L microscope (Nikon, Japan) or SLIDEVIEW^TM^ VS200 microscope (Olympus, Japan) and analyzed by the Image J software.

### Nissl’s staining

Nissl staining was executed as previously described [44]. Briefly, transverse sections (20 μm-thick) of spinal cords were stained with 0.1% cresyl violet for 6 min, rinsed in double-distilled water followed by ethanol (95%, 100%), cleared by xylene, and mounted. Image acquisition was finished with Ci-L microscope (Nikon, Japan) or SLIDEVIEW^TM^ VS200 microscope (Olympus, Japan) and analyzed by the Image J software.

### Luxol Fast Blue staining

Luxol Fast Blue staining was performed as previously described [45]. Briefly, the transverse sections (20 μm in thickness) of spinal cords were treated with 95% ethanol and dehydrated in 100% ethanol. Then, the sections were left in luxol fast blue solution at 56 °C for overnight and washed with 95% ethyl alcohol and distilled water. After differentiating with 0.05% lithium carbonate solution in 5–10 s, the sections were washed in 70% ethanol and double-distilled water. The sections were differentiated with 0.05% lithium carbonate solution and washed in 70% ethanol and double-distilled water until white matter and gray matter were in sharp contrast microscopically. Then the sections were washed in double-distilled water followed by ethanol (95%, 100%), cleared by xylene, and mounted. The images were captured via Ci-L microscope (Nikon, Japan) or SLIDEVIEW^TM^ VS200 microscope (Olympus, Japan) and analyzed by the Image J software.

### Western blotting

Briefly, after lysis with RIPA solution (P0013B, Beyotime) and incubation at 4 °C for 30 min, tissue lysates were centrifugated at 12,000 rpm for 20 min. Then the extracted proteins were mixed with loading buffer and boiled for 8–10 min at 100 °C. Subsequently, the samples were separated by 8–12% sodium dodecyl sulfate-polyacrylamide gel electrophoresis (SDS-PAGE) and transferred into nitrocellulose membranes (Life sciences, USA). After blocking with 5% skim milk for 1.5 h, the immunoblots were incubated with various primary antibodies at 4 °C for overnight. The primary antibodies included rabbit anti-YAP (1:1000, ab205270, Abcam), rabbit anti-p-YAP [1:1000, #13008, Cell Signaling Technology (CST)], rabbit anti-LATS1 (1:1000, #3477, CST), rabbit anti-p-LATS1 (1:1000, #9157, CST), rabbit anti-MST1 (1:1000, #3682, CST), rabbit anti-p-MST1/2 (1:1000, #49332, CST), rabbit anti-SAV1 (1:1000, #13301, CST), rabbit anti MOB1 (1:1000, #13730, CST), rabbit anti-HMGCS1 (1:1000, ab155787, Abcam) and mouse anti-MBP (1:1000, ab62631, Abcam). Mouse anti-β-actin (1:10,000, A5316, Sigma-Aldrich) or rabbit anti-GAPDH (1:5000, #2118, CST) was detected alongside the experimental samples as a loading control. Subsequently, the membranes were rinsed three times in TBST, and then incubated with proper secondary antibodies (1:5000, Pierce) for 2 h. The protein signals were detected by the ECL detection kit (1705061, Bio-Rad, USA), and analyzed by Quantity One software (Bio-Rad, USA).

### Immunostaining

For cultured cells, cells were washed three times by PBS and fixed in 4% PFA for 20 min. Then the permeabilization and blockade were done with 0.1% Triton X-100 in PBS containing 5% BSA for 0.5 h. Subsequently, the cells were incubated with multiple primary antibodies at 4 °C for overnight, washed three times with PBS, and then incubated with proper secondary antibodies (1:1000, Invitrogen) for 2 h. Primary antibodies contained mouse anti-GFAP (1:500, MAB360, Millipore) and rabbit anti-YAP (1:200, ab205270, Abcam). After being washed with PBS, cells were mounted.

For tissue section staining, after rinsed by PBS and fixed in 4% PFA for 30 min, 20 μm-thick transverse spinal cord sections were blocked in 5% BSA plus 0.3% Triton X-100 for 1 h, incubated with primary antibodies at 4 °C for overnight, and then with appropriate secondary antibodies (1:1000, Invitrogen) for 1 h. Primary antibodies included rabbit anti-YAP (1:200, ab205270, Abcam), rabbit anti-NeuN (1:500, ab177487, Abcam), rabbit anti-CD45 (1:500, ab10558, Abcam), rabbit anti-PH3 (1:500, #06-570, Millipore), rabbit anti-HMGCS1 (1:500, ab155787, Abcam), mouse anti-YAP (1:200, WH0010413M1, Sigma-Aldrich), mouse anti-GFAP (1:500, MAB360, Millipore), mouse anti-MBP (1:500, ab62631, Abcam), rabbit anti-NF (1:500, ab8135, Abcam), mouse anti-PH3 (1:500, ab14955, Abcam); and goat anti-Iba1 (1:500, ab5076, Abcam). The sections were stained with DAPI (1:1000, Sigma-Aldrich) to show the nucleus. Image acquisition was done by TCS SP8 confocal microscopy (Leica, Germany) or SLIDEVIEW^TM^ VS200 microscope (Olympus, Germany), and analyzed by the Image J software.

### Cell culture

Briefly, the cerebral neocortex of P1-P3 YAP^f/f^ and YAP^GFAP^-CKO mice were chopped and digested with 0.25% trypsin (Gibco) for 14–16 min at 37 °C, and then dissociated into a single-cell suspension by mechanical disruption. The cells were cultured in DMEM containing 10% fetal bovine serum (FBS, Gibco), on culture flasks coated with poly-l-lysine (0.1 mg/ml, Sigma-Aldrich). After cultured for 6–10 d, microglia and oligodendrocytes were removed by shaking for 4–6 h at 250 rpm. Subsequently, astrocytes were collected. The purity of GFAP^+^ cells in our system was more than 94%.

### RNA sequencing and functional enrichment analysis

Total RNA was obtained from cultured astrocytes (YAP^+/+^ and YAP^−/−^) by using the Trizol™ reagent (15596018, Invitrogen). The RNA samples with A260: A280 ratio > 1.8, A260: A230 ratio > 2.0 and the RNA integrity number > 7.0 were used for sequencing. The protocol has been described previously [41]. Briefly, differentially expressed genes were selected by the standard of fold change > 2 and adjusted *p*-value < 0.05. The heatmap analysis and the Kyoto encyclopedia of genes and genomes (KEGG) ontology enrichment analysis were made according to these differentially expressed genes. For KEGG enrichment analysis, a *p*-value < 0.05 was considered to be statistically significant.

### RNA extraction and qPCR

Total RNA was extracted from the spinal cords of mice or cultured cells. RNA was reversely transcribed into cDNA with a SuperScript™ One-Step Reverse Transcription Kit (#10928-034, Invitrogen). The expression levels of HMGCR, FDPS, and HMGCS1 mRNA were quantified using the iTaq™ Universal SYBR® Green Supermax (172-5122, Bio-Rad) on the real-time PCR detection system (Applied Biosystems, USA). GAPDH was used as the endogenous control. The relative mRNA expression levels were represented as ΔCt = Ct gene − Ct reference, and the fold change of gene expression was calculated by using the 2^−^^ΔΔCt^ method. The primers were described previously [46–49] and were synthesized by Sangon Biotech and presented as follows: *HMGCR*, 5′-AGT CAG TGG GAA CTA TTG CAC-3′ and 5′-TTA CGT CAA CCA TAG CTT CCG-3′; *FDPS*, 5′-AGG AGG TCC TAG AGT ACA ATG CC-3′ and 5′-TGA GGG AAG AGT CCA TGA TGT C-3′; *HMGCS1*, 5′-TGT TCT CTT ACG GTT CTG GC-3′ and 5′-AAG TTC TCG AGT CAA GCC TTG-3′; *GAPDH*, 5′-AGG TCG GTG TGA ACG GAT TTG-3′ and 5′-TGT AGA CCA TGT AGT TGA GGT CA-3′.

### Electron microscopy and quantitative analysis

The EAE mice were euthanized at 21 dpi. The spinal cords were first fixed with 2.5% glutaraldehyde (Sinopharm Chemical Reagent Co., Ltd) in PBS overnight, then washed three times by PBS and postfixed with 1% OsO4 (SPI-CHEM) in PBS for 1.5 h and then rinsed by PBS. After, the samples were first dehydrated in ascending dilution series of ethanol for 15 min at each step, and then dehydrated for 20 min by alcohol. Next, the samples were transferred to absolute acetone (Sinopharm Chemical Reagent Co., Ltd) for 20 min. The samples were then placed in 1:1 (1 h) and 1:3 (3 h) mixture of absolute acetone and the final Spurr resin mixture (SPI-CHEM) at room temperature, and then transferred to the final Spurr resin mixture overnight. After that, samples were embedded in Spurr resin and heated at 70 °C for more than 9 h, and then were sectioned in LEICA EM UC7 ultratome. Sections were stained by uranyl acetate and alkaline lead citrate (Sinopharm Chemical Reagent Co., Ltd) for 5–10 min, respectively, and observed in Hitachi Model H-7650 TEM. The analysis was performed with Image J. The G-ratios of myelinated fibers were calculated as the ratio of the axonal diameter to the myelinated fiber diameter as measured from different locations as described previously [50].

### Statistical analysis

All data values were expressed as mean ± SEM derived from at least three independent experiments. Statistical analysis was done with GraphPad Prism software. Student’s *t*-test was used for comparison between two groups, one-way ANOVA and two-way ANOVA with Bonferroni’s post-tests were performed for multiple groups. Details of each statistical test have been shown in the figure legends. A *p*-value of < 0.05 was considered to be statistically significant.

## Results

### YAP was upregulated and activated in astrocytes of EAE spinal cords through suppression of Hippo pathway kinases

**Fig. 1:**
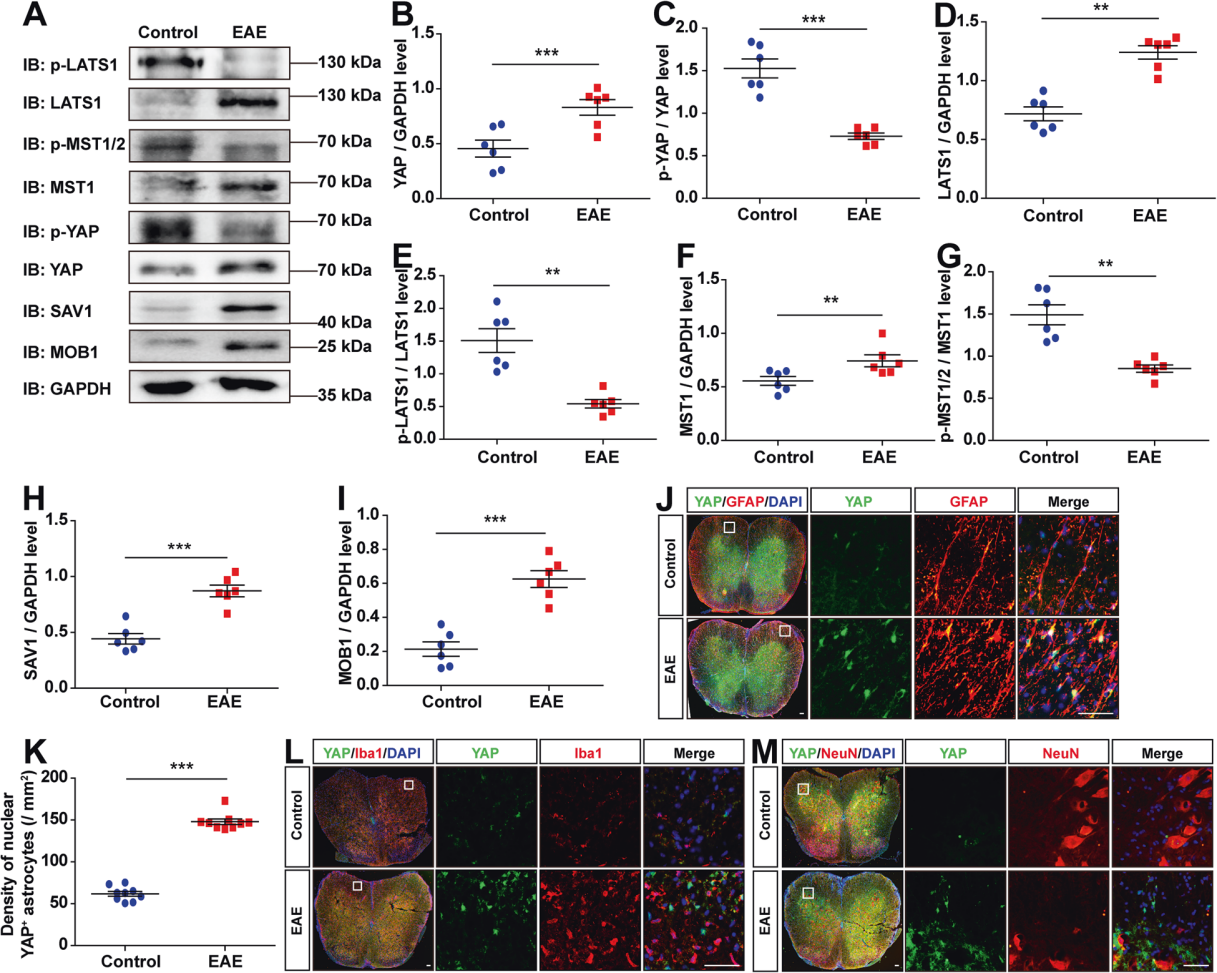
YAP was upregulated and activated in astrocytes through suppression of the Hippo pathway in EAE mice. **A** Western blot showed the p-LATS1, LATS1, p-MST1/2, MST1, p-YAP, YAP, SAV1 and MOB1 expression in the lumbar spinal cords of control and EAE mice. **B**–**I** Quantitative analysis of the YAP (**B**), p-YAP/YAP (**C**), LATS1 (**D**), p-LATS1/LATS1 (**E**), MST1 (**F**), (p-MST1/2)/MST1 (**G**), SAV1 (**H**) and MOB1 (**I**) protein levels as shown in **A** (*n* = 6, normalized to GAPDH). **J** Immunostaining of YAP (green) and GFAP (red) in the spinal cords of control and EAE mice. **K** Quantification of the density of nuclear YAP^+^ astrocytes as shown in **J** (*n* = 9). **L**, **M** Immunostaining of YAP (green) and Iba1 (red) (**L**) or YAP (green) and NeuN (red) (**M**) in the spinal cords of control and EAE mice. Scale bars, 50 μm. Data were mean ± SEM. Student’s *t*-test, **p < 0.01, ***p < 0.001.

To investigate the potential functions of YAP in MS, we first examined the expression pattern of YAP in EAE mice. Firstly, the EAE model was established successfully by MOG_35-55_ and a pertussis toxin boost injection (Supplementary Fig. S1). Interestingly, the expression of YAP was significantly increased in the lumbar spinal cords of EAE mice (Fig. 1A, B), however, p-YAP/YAP was decreased significantly (Fig. 1A, C), compared with that in control mice, indicating that YAP was upregulated and activated in the lumbar spinal cords of EAE mice. To further know whether activation of YAP in EAE mice was through suppression of Hippo kinases, several key kinases of the Hippo signaling pathway such as LATS1, MST1, SAV1, and MOB1 were examined, and the results showed that the expression levels of LATS1, MST1, SAV1, and MOB1 were significantly increased in the lumbar spinal cords of EAE mice, however, p-LATS1/LATS1 and (p-MST1/2)/MST1 levels were significantly decreased (Fig. 1A, D–I), indicating that activation of YAP in EAE was dependent on the suppression of Hippo signaling pathway.

To further explore the spatial expression pattern of YAP in the spinal cords of EAE mice, double immunostaining of YAP and several cell markers including GFAP (a marker of astrocytes), Iba1 (a marker of microglia), and NeuN (a marker of neurons) in control and EAE mice were performed. Interestingly, YAP was mainly expressed in GFAP^+^ astrocytes (Fig. 1J), not in Iba1^+^ microglial cells (Fig. 1L) or NeuN^+^ neurons (Fig. 1M), and was upregulated and located in the nuclear of GFAP^+^ astrocytes (Fig. 1J, K) in EAE mice. Overall, these results suggested that YAP was upregulated and activated in astrocytes of EAE spinal cords dependent on the suppression of Hippo signal pathway kinases.

### EAE was aggravated with earlier onset, severer inflammatory infiltration, and more loss of neurons in YAP^GFAP^-CKO mice

To further study the function of astrocytic YAP in EAE, YAP^GFAP^-CKO mice, which conditionally deleted YAP in astrocytes, were generated. In these YAP^GFAP^-CKO mice, YAP was efficiently knockout in the spinal cords, several brain regions including cerebellum, hippocampus, and cortex (Supplementary Fig. S2A, B), and primary cultured astrocytes (Supplementary Fig. S2C, E). However, YAP knockout did not significantly affect the number and distribution of neurons in the spinal cords (Supplementary Fig. S2F–I). As reported in our previous studies [41], the morphology and proliferation of astrocytes, and body weight were also comparable between YAP^f/f^ and YAP^GFAP^-CKO mice, and behavior tests such as rotarod performance, footprint, and pole test showed that YAP deletion did not affect the motor functions of mice significantly. These results suggested that the knockout of astrocytic YAP did not affect the development of the spinal cords and motor functions.

**Fig. 2:**
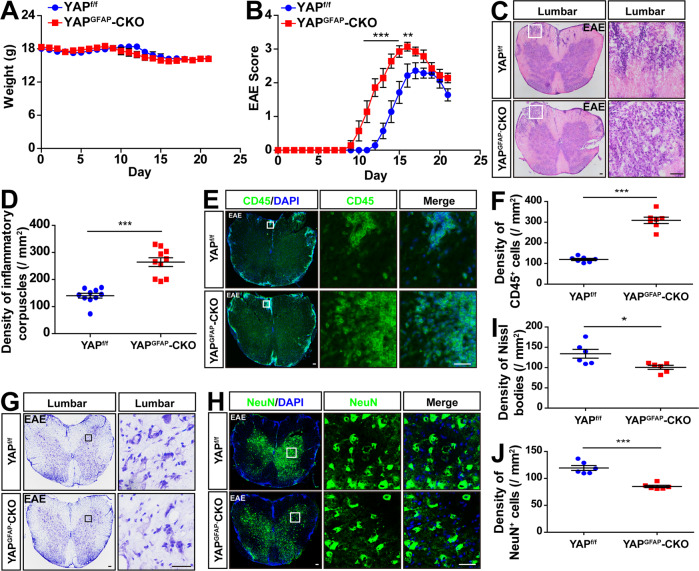
Knockout YAP in astrocytes aggravated EAE. **A** The body weight of YAP^f/f^ and YAP^GFAP^-CKO mice from 0 to 21 dpi during the EAE modeling process (*n* = 7, two-way ANOVA with Bonferroni’s post-tests). **B** The EAE score of YAP^f/f^ and YAP^GFAP^-CKO mice from 0 to 21 dpi during the EAE modeling process (*n* = 7, two-way ANOVA with Bonferroni’s post-tests). **C** Representative images of HE staining in the lumbar spinal cords of YAP^f/f^ and YAP^GFAP^-CKO EAE mice. **D** Quantification of the density of inflammatory corpuscles as shown in **C** (*n* = 10). **E** Immunostaining of CD45 (green) in the lumbar spinal cords of YAP^f/f^ and YAP^GFAP^-CKO EAE mice. **F** Quantification of the density of CD45^+^ cells as shown in **E** (*n* = 7). **G** Representative images of Nissl’s staining in the lumbar spinal cords of YAP^f/f^ and YAP^GFAP^-CKO EAE mice. **H** Immunostaining of NeuN (green) in the lumbar spinal cords of YAP^f/f^ and YAP^GFAP^-CKO EAE mice. **I** Quantification of the density of Nissl bodies as shown in **G** (*n* = 6). **J** Quantification of the density of NeuN^+^ cells as shown in **H** (*n* = 6). Scale bars, 50 μm. Data were mean ± SEM. Student’s *t*-test unless otherwise indicated, *p < 0.05, **p < 0.01, ***p < 0.001.

We next examined the roles of astrocytic YAP in EAE. In the body weight, there was no significant difference between YAP^f/f^ and YAP^GFAP^-CKO mice during the process of EAE modeling (Fig. 2A), however, knockout of astrocytic YAP showed significantly worse EAE scores at days 11–16 post injection (dpi) (Fig. 2B). The average time when the clinical score reached the peak in YAP^GFAP^-CKO mice was earlier than that in YAP^f/f^ mice. In YAP^f/f^ mice, the first clinical signs that the tip of the tail was limp were observed at 12–15 dpi, however, in YAP^GFAP^-CKO mice, the first clinical signs were observed at 9–11 dpi. These results suggested that the onset of EAE in YAP^GFAP^-CKO mice was advanced.

To know inflammatory infiltration in EAE mice, HE staining in the lumbar spinal cords of YAP^f/f^ and YAP^GFAP^-CKO EAE mice was performed. The density of inflammatory corpuscles was significantly increased in the spinal cords of YAP^GFAP^-CKO EAE mice, compared with that in YAP^f/f^ EAE mice (Fig. 2C, D), suggesting that knockout of astrocytic YAP aggravated the inflammatory infiltration of EAE mice. Moreover, the density of CD45^+^ cells was significantly increased in the spinal cords of YAP^GFAP^-CKO EAE mice, compared with that in YAP^f/f^ EAE mice (Fig. 2E, F). Furthermore, Nissl’s staining revealed that the density of Nissl bodies was decreased significantly in the spinal cords of YAP^GFAP^-CKO EAE mice (Fig. 2G, I). Finally, the density of NeuN^+^ cells was decreased significantly in the lumbar spinal cords of YAP^GFAP^-CKO EAE mice (Fig. 2H, J). These results suggested that deletion of astrocytic YAP aggravated EAE with earlier onset, severer inflammatory infiltration, and more loss of neurons.

### The demyelination of neurons was exacerbated in the spinal cords of YAP^GFAP^-CKO EAE mice

**Fig. 3:**
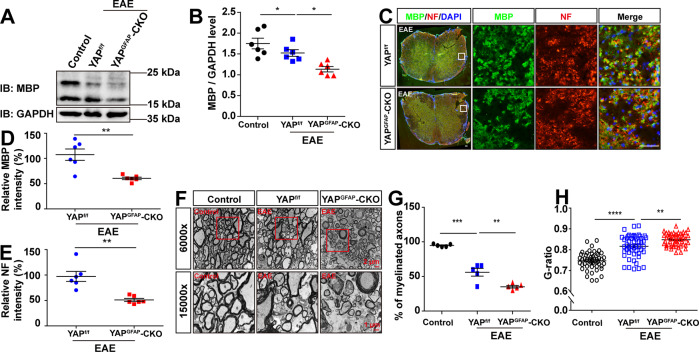
The demyelination of neurons was exacerbated in the spinal cords of YAP^GFAP^-CKO EAE mice. **A** Western blot showed the MBP expression in the lumbar spinal cords of control mice, YAP^f/f^ EAE mice, and YAP^GFAP^-CKO EAE mice. **B** Quantification of the total MBP protein level as shown in **A** (*n* = 6, normalized to GAPDH). **C** Immunostaining of MBP (green) and NF (red) in the lumbar spinal cords of YAP^f/f^ and YAP^GFAP^-CKO EAE mice. Scale bars, 50 μm. **D**, **E** Quantification of the relative intensity of MBP (**D**) and NF (**E**) as shown in (**C**) (normalized to control group, *n* = 6, Student’s *t*-test). **F** Representative the electron microscope images of control mice, YAP^f/f^ and YAP^GFAP^-CKO EAE mice at 21 dpi. Scale bars, 2 μm (low magnification) and 1 μm (high magnification). **G** Quantification of the percentages of myelinated axons in one field as shown in **F** (*n* = 5). **H** G-ratio as shown in **F** (*n* = 50). Data were mean ± SEM. One-way ANOVA with Bonferroni’s post-tests unless otherwise indicated, *p < 0.05, **p < 0.01, ***p < 0.001, ****p < 0.0001.

Given that demyelination is one of the characteristics of EAE [2, 51], we then tested whether the deletion of astrocytic YAP affected the demyelination of neurons in EAE mice. Western blot showed that the protein level of MBP (myelin basic protein, a marker protein of myelin) in the lumbar spinal cords of YAP^GFAP^-CKO EAE mice was decreased significantly, compared with that of YAP^f/f^ EAE mice (Fig. 3A, B). Furthermore, double immunostaining also showed that the intensity of MBP and NF (neurofilament heavy polypeptide) were decreased significantly in the lumbar spinal cords of YAP^GFAP^-CKO EAE mice (Fig. 3C–E). Finally, the electron microscope experiment further revealed that YAP^GFAP^-CKO EAE mice had severer demyelination phenotype in the lumbar spinal cords than that in YAP^f/f^ EAE mice, including the ratio of myelinated axons (Fig. 3F, G) and the G-ratio measurement (Fig. 3F, H). Taken together, these results indicated that astrocytic YAP prevented the demyelination of neurons in the spinal cords of EAE mice.

### The neuroinflammation was aggravated and the proliferation of astrocytes was reduced in the spinal cords of YAP^GFAP^-CKO EAE mice

**Fig. 4:**
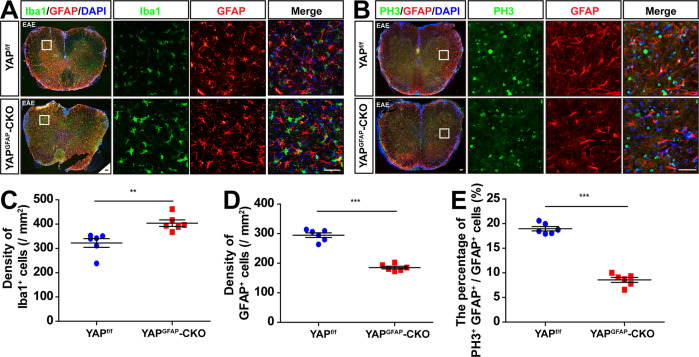
The neuroinflammation was aggravated and the proliferation of astrocytes was reduced in the spinal cords of YAP^GFAP^-CKO EAE mice. **A**, **B** Immunostaining of Iba1 (green) and GFAP (red) (**A**), and PH3 (green) and GFAP (red) (**B**) in the lumbar spinal cords of YAP^f/f^ and YAP^GFAP^-CKO EAE mice. **C**, **D** Quantification of the density of Iba1^+^ (**C**) and GFAP^+^ (**D**) cells in shown in **A** (*n* = 6). **E** Quantification of the percentage of PH3^+^ and GFAP^+^ cells over total GFAP^+^ cells in one field as shown in **B** (*n* = 6). Scale bars, 50 μm. Data were mean ± SEM. Student’s *t*-test, **p < 0.01, ***p < 0.001.

Above HE staining results showed that deletion of astrocytic YAP caused severer neuroinflammatory infiltration in EAE mice (Fig. 2C, D). Inflammatory cells in CNS under EAE conditions include microglia cells, astrocytes, and T/B cells [7–10], thus we next investigated whether deletion of astrocytic YAP affected the responses of these inflammatory cells by labeling their markers in the spinal cords of EAE mice. The density of Iba1^+^ microglia cells was increased significantly in the lumbar spinal cords of YAP^GFAP^-CKO EAE mice, compared with that of YAP^f/f^ EAE mice, while the density of GFAP^+^ astrocytes was significantly decreased (Fig. 4A, C, D). Furthermore, the proliferation of astrocytes was reduced in the lumbar spinal cords of YAP^GFAP^-CKO EAE mice, compared with that of YAP^f/f^ EAE mice (Fig. 4B, E). These results suggested that astrocytic YAP prevented the neuroinflammatory responses in EAE, and YAP was required for the astrocyte proliferation in the spinal cords of EAE mice.

### The cholesterol-synthesis pathway genes were downregulated in YAP^GFAP^-CKO EAE mice

**Fig. 5:**
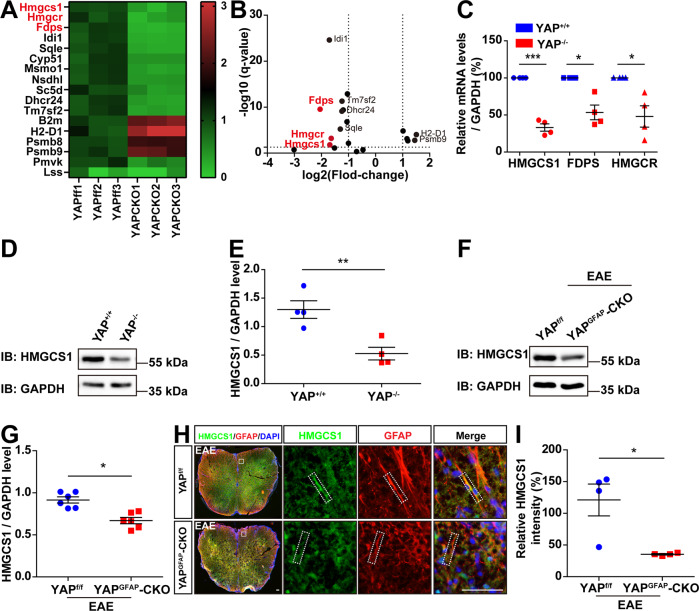
The cholesterol-synthesis pathway genes were downregulated in YAP^GFAP^-CKO EAE mice. **A**, **B** The heatmap and volcano plot of differential mRNAs of myelination-related genes sequenced in YAP^+/+^ and YAP^−/−^ astrocytes. **C** qPCR analysis showed the relative mRNA level of HMGCS1, FDPS and HMGCR in YAP^+/+^ and YAP^−/−^ astrocytes (*n* = 4, normalized to YAP^+/+^ control group, two-way ANOVA with Bonferroni’s post-tests). **D** Western blot showed the HMGCS1 expression in YAP^+/+^ and YAP^−/−^ astrocytes. **E** Quantification of HMGCS1 expression as shown in **D** (*n* = 4, normalized to GAPDH). **F** Western blot showed the HMGCS1 expression in the lumbar spinal cords of YAP^f/f^ and YAP^GFAP^-CKO EAE mice. **G** Quantification of the HMGCS1 expression as shown in **F** (*n* = 6, normalized to GAPDH). **H** Immunostaining of HMGCS1 (green) and GFAP (red) in the lumbar spinal cords of YAP^f/f^ and YAP^GFAP^-CKO EAE mice. **I** Quantification of the relative intensity of HMGCS1 as shown in **H** (normalized to YAP^f/f^ EAE group, *n* = 4). Scale bars, 50 μm. Data were mean ± SEM. Student’s *t*-test unless otherwise indicated, *p < 0.05, **p < 0.01, ***p < 0.001.

How does YAP deletion in astrocytes aggravate the demyelination in EAE? For this purpose, mRNA sequencing of YAP^+/+^ and YAP^−/−^ astrocytes were carried out. Interestingly, genes of the cholesterol-synthesis pathway, such as HMGCR, FDPS, and HMGCS1, were downregulated in YAP^−/−^ astrocytes, compared with that in YAP^+/+^ astrocytes (Fig. 5A, B). These genes have been reported to be involved in reparative synaptic plasticity and myelination in EAE [18]. qPCR analysis further confirmed the reduction of the mRNA level of HMGCS1, FDPS and HMGCR in YAP^−/−^ astrocytes (Fig. 5C). It has been reported that HMGCS1 is an important factor for cholesterol biosynthesis [52]. So, we further confirmed the decreased protein level of HMGCS1 in YAP^−/−^ astrocytes by western blot (Fig. 5D, E). These results indicated that YAP was required for the expression of cholesterol-synthesis genes such as HMGCS1 in astrocytes.

We next examined whether YAP was also involved in regulating the expression of cholesterol-synthesis genes in EAE mice. As expected, western blot revealed that the protein level of HMGCS1 was decreased in the lumbar spinal cords of YAP^GFAP^-CKO EAE mice, compared with that of YAP^f/f^ EAE mice (Fig. 5F, G). In addition, double immunostaining revealed that the expression of HMGCS1 was significantly decreased in astrocytes of the lumbar spinal cord of YAP^GFAP^-CKO EAE mice (Fig. 5H, I). Taken together, these results suggested that the cholesterol-synthesis pathway genes such as HMGCS1 might be downstream targets of YAP signaling, which might be involved in preventing demyelination in EAE mice.

### Upregulation of cholesterol-synthesis pathway promoted the expression of HMGCS1 and partially rescued the demyelination deficits in YAP^GFAP^-CKO EAE mice

**Fig. 6:**
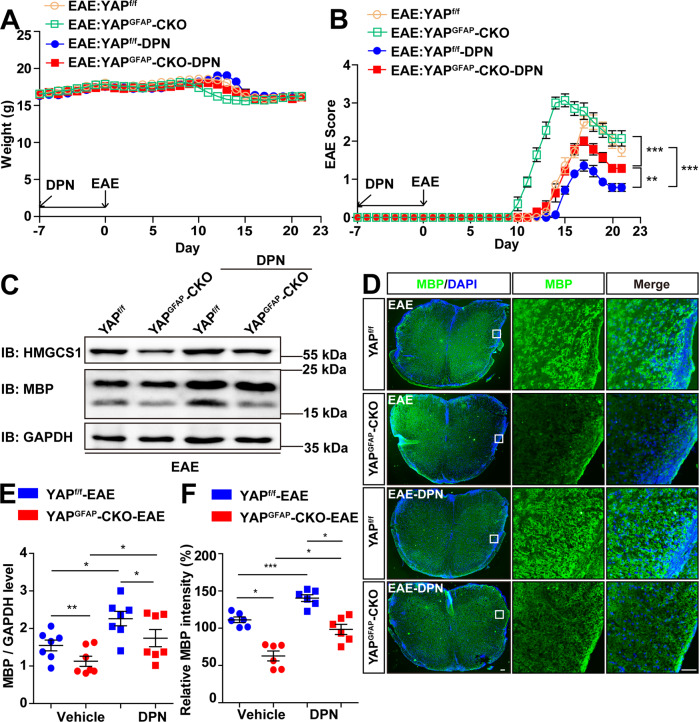
Upregulating cholesterol-synthesis pathways partially rescued the demyelination deficits in YAP^GFAP^-CKO EAE mice. **A** The body weight of YAP^f/f^ and YAP^GFAP^-CKO mice treated with vehicle or DPN from 0 to 21 dpi during the EAE modeling process (*n* = 7). **B** The EAE score of YAP^f/f^ and YAP^GFAP^-CKO mice treated with vehicle or DPN from 0 to 21 dpi during the EAE modeling process (*n* = 7). **C** Western blot showed the HMGCS1 and MBP expression in the lumbar spinal cords of YAP^f/f^ and YAP^GFAP^-CKO EAE mice treated with vehicle or DPN. **D** Immunostaining of MBP (green) in the lumbar spinal cords of YAP^f/f^ and YAP^GFAP^-CKO EAE mice treated with vehicle or DPN. **E** Quantification of the MBP level as shown in **C** (*n* = 7, normalized to GAPDH). **F** Quantification of the relative intensity of MBP as shown in **D** (normalized to YAP^f/f^ EAE group, *n* = 6). Scale bars, 50 μm. Data were mean ± SEM. Two-way ANOVA with Bonferroni’s post-tests, *p < 0.05, **p < 0.01, ***p < 0.001.

It has been reported that ERβ-ligand treatment could induce an increase in cholesterol-synthesis gene expression in oligodendrocytes during remyelination [43]. Therefore, we next examined whether the application of diarylpropionitrile (DPN) (an ERβ-ligand, to upregulate the expression of HMGCS1) rescued the demyelination deficits in YAP^GFAP^-CKO EAE mice. As expected, we found that DPN treatment significantly improved the functional recovery in YAP^GFAP^-CKO EAE mice, compared with that in vehicle-treated YAP^GFAP^-CKO EAE mice. However, the EAE score of DPN-treated-YAP^GFAP^-CKO EAE mice was significantly higher than that of DPN-treated-YAP^f/f^ EAE mice (Fig. 6A, B). Furthermore, western blot and immunostaining revealed that DPN treatment could partially rescue the decrease of HMGCS1 and MBP expression in the lumbar spinal cords of YAP^GFAP^-CKO EAE mice (Fig. 6C–F), compared with that in vehicle-treated YAP^GFAP^-CKO EAE mice. Overall, these results suggested that upregulation of cholesterol-synthesis pathways promoted the expression of HMGCS1 and then partially rescued the demyelination deficits in YAP^GFAP^-CKO EAE mice.

### Activation of YAP signaling by XMU-MP-1 promoted the expression of HMGCS1 in astrocytes, prevented the demyelination and neuroinflammation, and improved the functional recovery in EAE mice

**Fig. 7:**
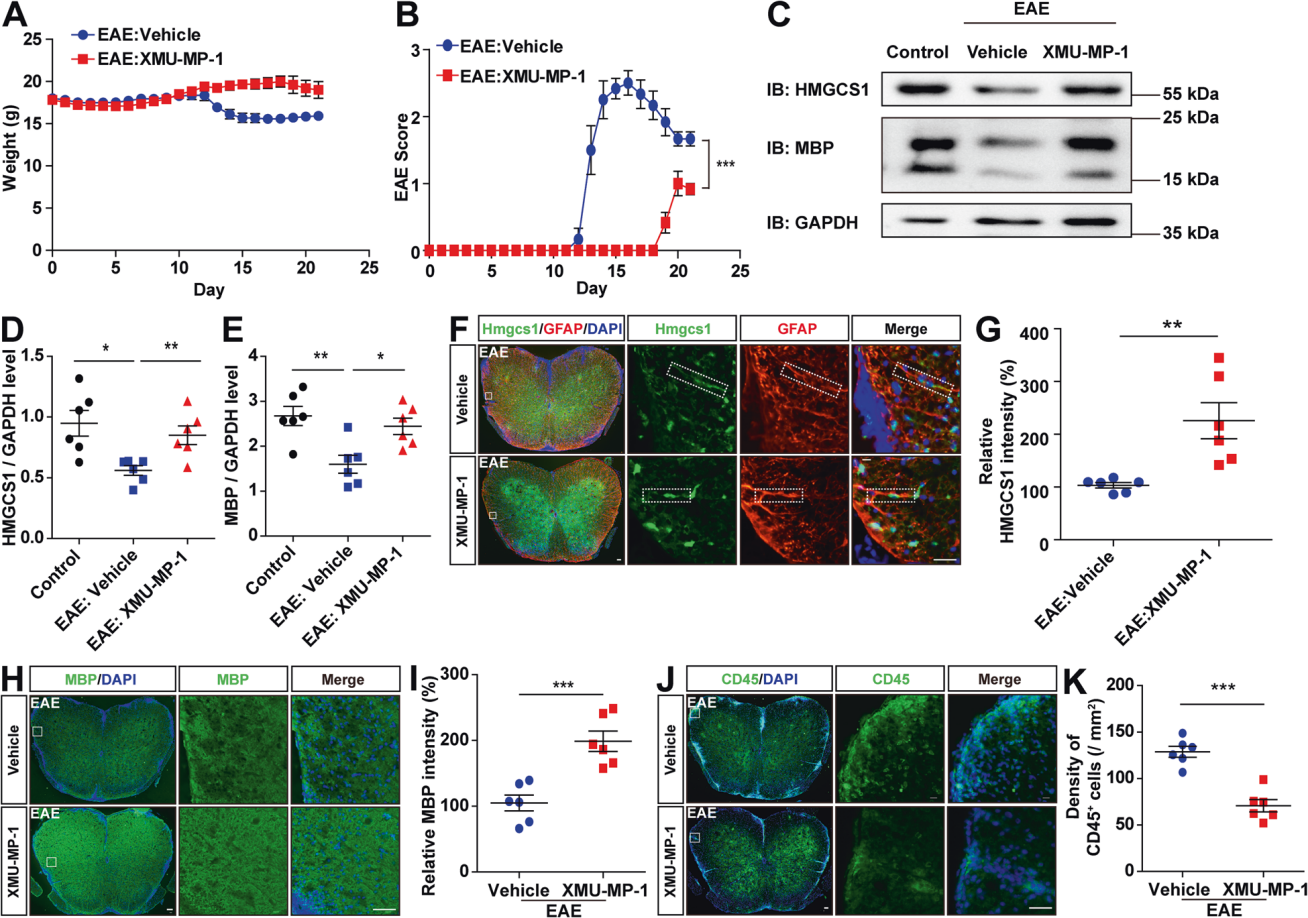
The activation of YAP signaling prevented the demyelination and neuroinflammation, and improved the functional recovery of EAE mice. **A** The body weight of vehicle-treated mice and XMU-MP-1-treated mice from 0 to 21 dpi during the EAE modeling process (*n* = 6, two-way ANOVA with Bonferroni’s post-tests). **B** The EAE score of vehicle-treated mice and XMU-MP-1-treated mice 0–21 dpi during the EAE modeling process (*n* = 6, two-way ANOVA with Bonferroni’s post-tests). **C** Western blot showed the HMGCS1 and MBP expression in the lumbar spinal cords of vehicle-treated EAE mice and XMU-MP-1-treated EAE mice. **D**, **E** Quantification of HMGCS1 (**D**) and MBP (**E**) protein level as shown in **C** (*n* = 6, normalized to GAPDH, one-way ANOVA with Bonferroni’s post-tests). **F** Immunostaining of HMGCS1 (green) and GFAP (red) in the lumbar spinal cords of vehicle-treated EAE mice and XMU-MP-1-treated EAE mice. **G** Quantification of the relative intensity level of HMGCS1 as shown in **F** (normalized to vehicle EAE group, *n* = 6). **H** Immunostaining of MBP (green) in the lumbar spinal cords of vehicle-treated EAE mice and XMU-MP-1-treated EAE mice. **I** Quantification of the relative intensity of MBP as shown in **H** (normalized to vehicle EAE group, *n* = 6). **J** Immunostaining of CD45 (green) in the lumbar spinal cords of vehicle-treated EAE mice and XMU-MP-1-treated EAE mice. **K** Quantification of the density of CD45^+^ cells as shown in **J** (*n* = 6). Scale bars, 50 μm. Data were mean ± SEM. Student’s *t*-test unless otherwise indicated, *p < 0.05, **p < 0.01, ***p < 0.001.

We next tested whether activation of YAP signaling in astrocytes could treat EAE mice. XMU-MP-1 (MST1/2 inhibitor) was used to inhibit the Hippo signaling pathway to activate YAP, and then was applied to treat EAE mice. Indeed, XMU-MP-1 significantly improved the functional recovery of EAE mice, compared with that in the control treatment (Fig. 7A, B). As expected, western blot revealed that the protein levels of HMGCS1 and MBP in the lumbar spinal cords were increased significantly in the spinal cords of XMU-MP-1-treated EAE mice (Fig. 7C–E). Double immunostaining revealed that the expression of HMGCS1 was increased significantly in astrocytes of the lumbar spinal cords of XMU-MP-1-treated EAE mice (Fig. 7F, G). Furthermore, immunostaining of MBP and CD45 revealed that XMU-MP-1 prevented the demyelination and neuroinflammation in the spinal cords of EAE mice (Fig. 7H–K). These results indicated that activation of YAP promoted the expression of HMGCS1 in astrocytes, prevented demyelination and neuroinflammation, and improved the functional recovery of EAE mice.

## Discussion

**Fig. 8:**
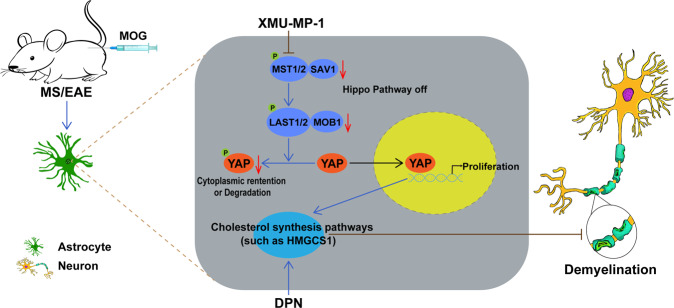
A working model of astrocytic YAP’s function in EAE mice. In the spinal cords of EAE mice, YAP is upregulated and activated in astrocytes by suppression of Hippo signaling including MST1/2, SAV1, LATS1/2 and MOB1, and promotes the proliferation of astrocytes and induces the expression of cholesterol-synthesis genes such as HMGCS1, which prevents the neuroinflammation and demyelination. XMU-MP-1, an inhibitor of MST1/2; DPN, an ERβ-ligand.

In our study, we provide evidence for YAP’s function in astrocyte proliferation, demyelination, and neuroinflammation in EAE mice, and propose its working model (Fig. 8). In this model, YAP is upregulated and activated in astrocytes of EAE mice dependent on suppression of the Hippo pathway including MST1, SAV1, LATS1, and MOB1, and promotes the proliferation of astrocytes and induces the expression of cholesterol-synthesis genes such as HMGCS1, which contributes to preventing the demyelination in the spinal cords of EAE mice.

Recent reports have shown that YAP is upregulated and activated in astrocytes through suppression of the Hippo pathway after spinal cord injury, and promotes glial scar formation and neural regeneration, and improves functional recovery of mice after SCI [41]. Also, our recent research has shown that astrocytic YAP could prevent the demyelination and neuroinflammation of the retina and optic nerve of EAE through upregulating TGF-β signaling [53]. Interestingly, consistent with these previous studies, present studies showed that in EAE mice, YAP was also upregulated and activated through suppression of Hippo pathway in astrocytes, not in microglia or neurons of spinal cords (Fig. 1), which suggest that astrocytic YAP may be activated and play some conservative roles under different pathological conditions.

We also notice that the number of microglia was increased, while the number of astrocytes was decreased in the spinal cords of YAP^GFAP^-CKO EAE mice. Because YAP is not expressed in microglia cells [29], thus it may be secondary effects of YAP knockout in astrocytes. Our previous studies have shown that both cytokines and chemokines were increased in YAP^−/−^ astrocytes [29], thus it is very likely that YAP^−/−^ astrocytes may secret some cytokines such as TNF-α to promote the activation and the proliferation of microglia. The reduced number of astrocytes may attribute to decreased proliferation of astrocytes, as indicated by the proliferating marker PH3 (Fig. 4B, E). Previous research has reported that YAP promotes the differentiation and proliferation of astrocytes [34], and ablation of astrocytic YAP impairs the formation of glial scar and increases the size of injury because of the decrease of astrocytic proliferation after SCI [41]. Our present studies were consistent with these previous studies. Thus, the reduction of proliferation of astrocytes by YAP knockout resulted in decreased astrocytes number in EAE mice, which might contribute to aggravating EAE.

In the adult brain, peripheral cholesterols do not cross the blood–brain barrier, thus most cholesterols must be synthesized in the brain [24, 25]. Studies have shown that in the adult brain, cholesterols are mainly synthesized in astrocytes and transported to oligodendrocytes and neurons [18]. In our studies, several evidence lines support that cholesterol-synthesis genes may be target genes of YAP in astrocytes in EAE. Firstly, mRNA sequencing revealed that cholesterol-synthesis genes such as HMGCS1 were decreased in YAP^−/−^ astrocytes (Fig. 5A, B). Secondly, cellular and biochemical experiments further confirmed that HMGCS1 was decreased in YAP^−/−^ astrocytes, and more obviously downregulated in spinal cords of YAP^GFAP^-CKO EAE mice (Fig. 5C–I). Finally, DPN, an ERβ-ligand, which upregulated the expression of HMGCS1, could partially rescue the demyelination deficits in YAP^GFAP^-CKO EAE mice, and improve functional recovery of YAP^GFAP^-CKO EAE mice (Fig. 6). These results strongly indicate that YAP induces the expression of cholesterol-synthesis genes to prevent demyelination of neurons in EAE mice. In fact, in the liver, YAP can stimulate the transcription of sterol regulatory element-binding proteins (SREBP-1c and SREBP-2), which are the promoters of the fatty acid synthase and HMGCR, and promote hepatocyte lipogenesis and cholesterol synthesis by interacting with them [39]. Other studies also have shown that the core member of the Hippo pathway LATS2 inhibits SREBP and suppresses hepatic cholesterol accumulation [40]. Consistent with these studies, our results revealed that XMU-MP-1 could activate YAP signaling and prevent demyelination, and improved the functional recovery of EAE mice, which might be due to the promotion of cholesterol synthesis in astrocytes. These results indicate that Hippo/YAP pathway plays a key role in cholesterol homeostasis in astrocytes or liver cells. Further experiments such as chromatin immunoprecipitation assays should be conducted to further explore how YAP regulates the expression of cholesterol-synthesis genes such as HMGCS1 in astrocytes of EAE mice.

In summary, our study identifies unrecognized functions of astrocytic YAP in the prevention of demyelination and neuroinflammation and reveals a novel pathway of YAP-HMGCS1 for cholesterol synthesis in astrocytes during EAE, which may help to develop new therapeutics for MS.

## Supplementary information

Supplementary Information

## Data Availability

The data that support the finding of this study are available upon request from the corresponding author. The raw sequence data have been deposited in the Genome Sequence Archive under accession number CRA004941.
