# Supplementary material for: Astrocytic YAP prevents the demyelination through promoting expression of cholesterol synthesis genes in experimental autoimmune encephalomyelitis
Source: Cell Death Dis. 2021 Oct 5;12(10):907. doi: 10.1038/s41419-021-04203-8 (PMC8492624; doi:10.1038/s41419-021-04203-8)
Supplement: Supplementary file 1 — Supplementary Information [file 41419_2021_4203_MOESM1_ESM.docx]

**Supplementary Information**

**Figure S1. Establishment of EAE model.**

(**A**) Quantification of the body weight of control and EAE mice from day 0 to day 21 during the EAE modeling process (n=8, two-way ANOVA with Bonferroni’s post-tests). (**B**) The EAE score of control and EAE mice from day 0 to day 21 during the EAE modeling process (n=8, two-way ANOVA with Bonferroni’s post-tests). (**C**) Representative images of HE staining in the lumbar spinal cords of control and EAE mice. (**D**) Quantification of the density of inflammatory corpuscles as shown in (**C**) (n=6). (**E**) Immunostaining of CD45 (red) in the lumbar spinal cords of control and EAE mice. (**F**) Quantification of the density of CD45^+^ cells as shown in (**E**) (n=6). (**G**) Representative images of Nissl's staining in the lumbar spinal cords of control and EAE mice. (**H**) Quantification of the density of Nissl bodies of control and EAE mice as shown in (**G**) (n=6). (**I**) Immunostaining of NeuN (green) in the lumbar spinal cords of control and EAE mice. (**J**) Quantification of the density of NeuN^+^ cells as shown in (**I**) (n=6). (**K**) Immunostaining of MBP (green) and NF (red) in the lumbar spinal cords of control and EAE mice. (**L**) Representative images of Luxol Fast Blue staining in the lumbar spinals cord of control and EAE mice. Scale bars, 50 μm. Data were mean ± SEM. Student’s *t*-test unless otherwise indicated, compared with control mice, ^***^*p < 0.001*.


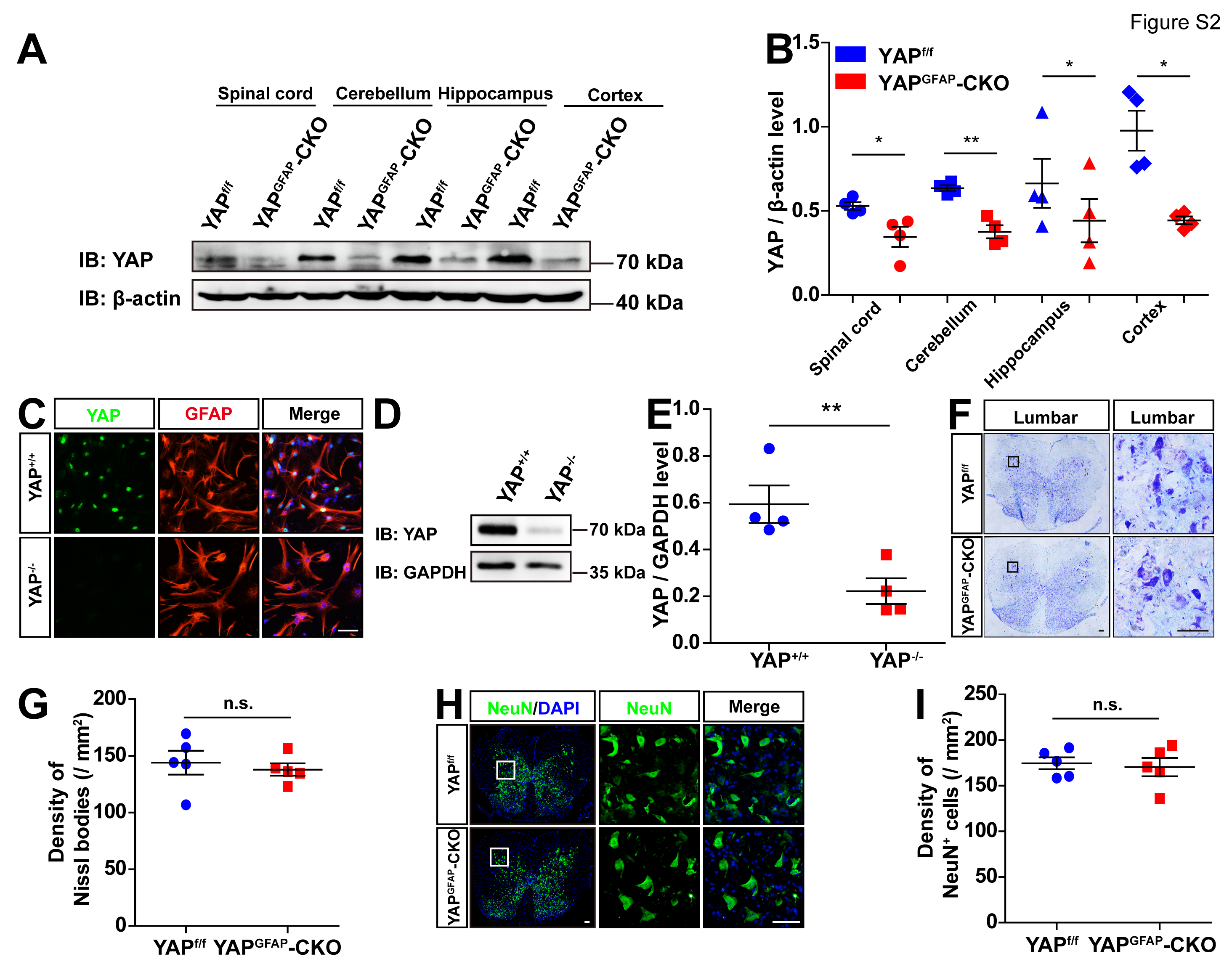


**Figure S2. Identification and normal spinal cord development of YAP^GFAP^-CKO mice.**

**(A)** Western blot detected the YAP expression in the spinal cord, cerebellum, hippocampus and cortex of 2-month YAP^f/f^ and YAP^GFAP^-CKO mice. (**B**) Quantification of the YAP expression as shown in (**A**) (n=4, normalized to β-actin). (**C**) Immunostaining of YAP (green) and GFAP (red) in cultured YAP^+/+^ and YAP^-/-^ astrocytes. Scale bars, 25 μm. (**D**) Western blot showed the YAP expression in cultured YAP^+/+^ and YAP^-/-^ astrocytes. (**E**) Quantification of the YAP expression as shown in (**D**) (n=4, normalized to GAPDH). (**F**) Representative images of Nissl's staining in the lumbar spinal cords of 2-month YAP^f/f^ and YAP^GFAP^-CKO mice. Scale bars, 50 μm. (**G**) Quantitation of the density of Nissl bodies as shown in (**F**) (n=5). (**H**) Immunostaining of NeuN (green) and DAPI (blue) in spinal cords of 2-month YAP^f/f^ and YAP^GFAP^-CKO mice. Scale bars, 50 μm. (**I**) Quantitation of the density of NeuN^+^ cells as shown in (**H**) (n=5). Data were mean ± SEM. Student’s *t*-test, compared with YAP^f/f^ group, ^*^*p* *< 0.05*, ^**^*p* *< 0.01*, n.s., not significant (*p > 0.05*).
